# Implementing multicomponent, eHealth-based behaviour change support within a dietary intervention trial improves adherence to study-related behaviours in healthy young adults

**DOI:** 10.1186/s40795-023-00798-7

**Published:** 2023-11-21

**Authors:** Anna Worthington, Nicola Gillies, Rina Hannaford, Rajshri Roy, Andrea Braakhuis

**Affiliations:** 1 Discipline of Nutrition, School of Medical Sciences, Faculty of Medical and Health Sciences, The University of Auckland, Auckland, New Zealand; 2 Bioinformatics & Statistics Team, AgResearch Ltd, Palmerston North, New Zealand

**Keywords:** Behaviour change techniques, Adherence, eHealth, Health behaviour

## Abstract

**Background:**

Behaviour change science is proposed to improve participant retention and enhance the validity of trials. However, researchers seldom systematically consider and implement behaviour change strategies within trials for this purpose. The objective of this article is to evaluate how an eHealth behaviour change support (BCS) program enhances young adults’ adherence to behaviours required within a dietary intervention.

**Methods:**

The Nine Principles framework was used to develop BCS to implement across both arms of a 10-week randomised parallel-group intervention to enhance adherence to (i) eating healthily and (ii) reporting dietary intake. Key components of the BCS included access to a dietitian-led Facebook group, text reminders, and food delivery. Effectiveness was measured using the following analyses of the 78 participants who completed the study; pre-post change in targeted dietary habits over time using a subscore of the Healthy Diet Habits Index, questionnaire to assess change in perception of barriers to eating healthily over time, Facebook group engagement, and impact evaluation of the BCS. Participants received a dietary reporting score out of 100 to assess adherence across the 10 weeks.

**Results:**

The total Healthy Diet Habits Index subscore out of 16 significantly increased from baseline to week 10 (10.6 ± 2.6 to 11.2 ± 2.6, *p* value < 0.05), driven primarily by an increase in vegetable consumption. Overall adherence to reporting was high across the 10 weeks, with the total population mean reporting score 90.4 ± 14.6 out of 100. Relatively low Facebook engagement was observed. Adding objects to the environment, prompts/cues and removing reward appeared to be effective components of the BCS for enhancing adherence to the target behaviours.

**Conclusion:**

Using a behaviour change framework to support the design of randomised trials is a promising way to enhance participant adherence to study requirements that are typically considered burdensome, such as dietary reporting. It also enables researchers to identify and replicate effective components of BCS, including behaviour change techniques and modes of delivery. Further research into the use of different behaviour change frameworks for this purpose is warranted.

**Trial registration:**

ClinicalTrials.gov Identifier: NCT04869163; https://clinicaltrials.gov/ct2/show/NCT04869163. (03/05/2021).

**Supplementary Information:**

The online version contains supplementary material available at 10.1186/s40795-023-00798-7.

## Background

Nutrition-related randomised controlled trials conducted in free-living populations provide key evidence for public health dietary guidelines and medical nutrition therapy guidelines to prevent and manage non-communicable diseases. Substantial funds and resources are invested into these trials every year [1]. Studying diets is complex in that there are many variables that can contribute to observed results, from the quantity and quality of food categories consumed over months, to the interactions of nutrients within a single meal [2]. To account for these food-related factors, diets must be accurately monitored. This is a current challenge of nutrition research, as dietary assessment methods can involve inherent error, and be difficult and time-consuming for participants to complete [3, 4]. Monitoring dietary intake is also essential for assessing the level to which participants adhere to a dietary pattern within a study, as poor adherence can result in a smaller observed effect size [5, 6]. Low participant adherence and high attrition rate is an undesirable, yet common challenge within nutrition trials [3, 7, 8]; it can make it difficult to draw meaningful conclusions about the effect of the dietary intervention, which in turn can limit trial validity and result in resource waste [4, 9].

Researchers frequently use strategies, such as prompts and monetary incentives, to encourage study-related behaviours like filling in questionnaires or attending clinic visits [10]. In behaviour change science, these strategies are known as behaviour change techniques (BCTs); they are considered the ‘active components’ that bring about change in a behaviour [11]. For example, explaining to a participant how to fill out a survey would be considered as the BCT ‘instruction on how to perform the behaviour’ [11]. Clear, consistent definitions of BCTs are provided through taxonomies, such as the BCT Taxonomy (v1), which allows identification of effective components within an intervention to advance the field of behaviour change [11]. Currently behaviour change science, such as the use of BCTs, is implicitly used in nutrition trials to improve participant adherence and retention; however, seldom are strategies to support participant adherence systematically implemented or theoretically informed, making it challenging to identify and replicate effective components [10, 12].

A recent proposal has been to incorporate behaviour change science in a more systematic way to improve the design of trials for improved participant retention, and enhanced validity and replicability of research [1, 10] For example, implementation science frameworks have been applied to the clinical trial context to enhance enrolment [13]. However, beyond enrolling there are extensive study-related behaviours participants are required to perform, particularly in nutrition trials, such as adhering to a prescribed diet, completing dietary assessments, and attending clinic visits. Hence, in this emerging area of research we investigate the use of behaviour change frameworks as an effective system for enhancing adherence to behaviours required within a nutrition trial.

The Protein Diet Satisfaction (PREDITION) trial investigated the effects of moderate lean red meat consumption as part of a balanced diet. It was a randomised parallel-group trial in which 80 young adults (aged 18 − 35 years) followed a diet containing pasture-fed red meat or vegetarian alternatives for 10 weeks [14]. Within this trial, two key participant behaviours were identified within the study requirements, which included (i) consuming a basal, healthy, vegetarian diet, and (ii) reporting dietary intake using a smartphone application. The first behaviour aimed to ensure the background dietary pattern of both arms was similar and representative of a balanced diet, as per the research question. The second behaviour was required to enable researchers to accurately assess participant adherence to the intervention, an often poorly reported but crucial element of trials [3]. A user-centred, theory-based behaviour change framework was used to develop Behaviour Change Support (BCS) for these behaviours within the PREDITION trial. We use the term BCS to refer to the strategies integrated within a study protocol and standardised across intervention groups to support adherence to behaviour, as opposed to a behaviour change intervention that targets behaviour as the primary outcome and differs across groups within a randomised trial. This study evaluated the effectiveness of an eHealh-based BCS program at enhancing participant adherence to the study-related behaviours of (i) eating a healthy basal diet and (ii) dietary reporting using a smartphone app within the PREDITION trial. The primary outcome of the current manuscript is to assess the effectiveness of the BCS in terms of,

1. Pre-post change in targeted dietary behaviours over time,
2. Change in perception of barriers to healthy eating over time,
3. Adherence to dietary reporting,
4. Facebook group engagement and,
5. Impact evaluation of the BCS.

## Methods

### Study design

The detailed PREDITION trial protocol has been previously published [14]. In short, 80 young adults were recruited as 40 household pairs, and arranged into eight groups, where groups reflect the chronological start time of participants. Participants on both intervention arms were required to change from an omnivorous diet to a basal, healthy vegetarian diet, aside from their allocated intervention protein. Participants were asked to report their dietary intake daily using images (Tuesday to Saturday) and direct entry (Sunday and Monday) into a smartphone application, titled ‘Easy Diet Diary’ (version 6.0.28, Xyris Software Pty Ltd, 2020) to assess adherence to the intervention diet [15].

Electronic health- (eHealth-) based, dietitian-led BCS was implemented across both arms to assist participants over the duration of the study to (i) achieve basal healthy dietary behaviours based on the New Zealand Ministry of Health Eating and Activity Guidelines [16] and (ii) report dietary intake using the Easy Diet Diary. The Nine Principles framework [17] was used to develop the BCS. Key steps of this process included a literature review to define current dietary behaviours of young adults; target audience surveys of young adults to identify barriers and enablers to these behaviours as well as the behaviour of reporting dietary intake; the Theory of Planned Behaviour, which describes how an individual’s decision to engage in a behaviour is governed by their intention to do so [18, 19], was then used to map the identified barriers and enablers to select effective levers of change; target audience focus groups were conducted for input on the draft BCS strategy; and the resulting BCS was piloted.

The final BCS strategy consisted of multiple BCTs, which were delivered via text messages and nine private groups on Facebook (Meta, 2020), each with up to 10 participants. Facebook and texts were selected as modes of delivery as they were deemed acceptable by the focus groups during development and feasible by researchers. The BCTs targeting each behaviour have been summarised in Table 1. Participants received two texts and 1–3 Facebook posts per week. The trial design meant participants met face-to-face with the dietitians for one screening visit and two clinic visits for data collection over 12 weeks. Additional to receiving 3 portions of their allocated protein a week, participants also received a weekly meal kit containing 3 vegetarian dinners for the study duration. Full details on the development of the BCS have been published elsewhere [20]. Of note, participants in group 7 were split across two Facebook groups, 7a and 7b, due to covid-related disruptions, but no changes in the content or delivery of the BCS occurred during the trial to standardise the support across both arms. The Template for Intervention Description and Replication framework was used to ensure elements of the BCS were sufficiently reported (see Additional file 1) [21].

**Table 1:** Behaviour change techniques used to support young adults to report dietary intake and eat healthily

**BCT**^**a**^** Code**	**BCT Name**	**Description of BCT**	
		**Reporting Dietary Intake**	**Eating Healthily**
**3.1**	Social support (unspecified)	Requirement to participate as a dining partner who you are accountable to	Connected to other participants and one dietitian via private Facebook group and Facebook Messenger chat (Facebook); Ad-libitum access to for the 10 weeks
**4.1**	Instruction on how to perform a behaviour	A4 information sheet^b^ emailed to participants at baseline on how to use the Easy Diet Diary App	Verbal instruction given in 4 Facebook videos^b^ by dietitians over 10 weeks regarding healthy eating; instructional Facebook posts on how to plan, buy, and cook healthily; hardcopy cookbook^b^ provided at first clinic visit
**6.1**	Demonstration of the behaviour	In-person demonstration of how to use the app at first clinic visit	Healthy cooking videos^b^ shared in Facebook groups
**7.1**	Prompts/cues	Three text reminders^b^ to report intake sent per week	Participants were asked via Messenger to turn on notifications for Facebook to receive prompts regarding healthy eating
**9.1**	Credible source	Information and instruction delivered by two New Zealand Registered Dietitians (mostly via Facebook, minimal instruction also given at screening and initial clinic visit)	
**2.2**	Feedback on behaviour	If participants fell below the level of necessary reporting for each 3- or 4-day period they received a text^a^ telling them this and to increase their reporting	NA
**8.1**	Behavioural practice/rehearsal	Participants were required to practice entering a manual and photographic entry into the app prior to beginning the intervention	NA
**14.3**	Remove reward	Participants were told from screening if them or their dining partner’s level of reporting was consistently insufficient they would stop receiving the free food and be removed from the trial	NA
**1.1**	Goal setting (behaviour)	NA	Participants prompted to set dietary goals every 3^rd^ week
**1.2**	Problem solving	No	Dietitians gave examples of barriers and how to solve them, followed by prompting participants to do the same in the 4 Facebook videos^b^
**5.1**	Information about health consequences	No	Information given on the long- and short-term health consequences of the dietary sub-behaviours targeted in the Facebook videos and posts^b^
**5.3**	Information about social and environmental consequences	No	Information given on the social and environmental consequences of the dietary sub-behaviours targeted in the Facebook videos and posts^b^, such as cost-saving benefits
**12.5**	Adding objects to the environment	No	Receiving weekly food boxes containing 3 vegetarian dinners

^a^*BCT* Behaviour Change Technique

^b^Resources including Facebook videos and posts, information sheets, cookbook, and text message templates are available from corresponding author upon reasonable request

### Recruitment and eligibility criteria

A total of 40 pairs of individuals aged 18–35 years who cohabitate and typically share evening meals were recruited. Recruitment was advertised with posters placed around the University of Auckland and using social media websites and tools. Potential participants filled out an online screening questionnaire, which was followed up by a screening clinic visit at which the consent form was signed by willing participants. All participants were required to be omnivores, i.e., in the last 2 months they consumed at least 2–3 meals per week which included meat of any description (red or white-fleshed meat, including fish), and were willing to consume both red meat and meat alternatives for the trial. Those with chronic health conditions, obesity (BMI ≥ 30 kg/m^2^), hyperlipidaemia, history of anosmia and ageusia (issues with smell and taste), use of medications (except for occasional nonsteroidal anti-inflammatory drugs and antihistamines), or recreational drugs, or those who smoke tobacco were excluded from participating. The Three-Factor Eating Questionnaire (TFEQ) evaluates cognitive and behavioural domains of eating [22]; a self-administered short-form (TFEQ-18) was completed online during participant screening to exclude participants with potentially disordered eating behaviours (TFEQ ≥ 0.75). Women were required to confirm they were neither pregnant nor intending to become pregnant during the trial. Potential participants who use dietary supplements were asked to abstain for the month before the beginning of the study. Participants were required to own a mobile phone with a camera and have a Facebook profile, or be willing to set one up, to access the BCS.

### Outcome measures

#### Change in dietary behaviours

A subscore of the Healthy Diet Habits Index (HDHI) was used to assess participants’ change in targeted dietary behaviours over time. The original HDHI, validated in a New Zealand adult population, is comprised of 15-items which assess the extent to which dietary behaviours align with dietary guidelines [23]. During the development of the BCS specific dietary behaviours that needed to change in the target population were identified. Hence, four items from the original HDHI relating to these behaviours were selected as a subscore (Table 2) [20]. These included daily intake of fruits and vegetables, weekly intake of sugar-sweetened beverages (SSBs), and types of breads or cereals consumed. The Short Food Frequency Questionnaire for New Zealand Adults [24] was completed online by participants at baseline and week 10, which was used by study dietitians to calculate their HDHI subscores. Each behaviour received a score between 0–4, with a higher score indicating the behaviour aligning with the 2020 Ministry of Health Eating Guidelines, such as meeting the recommended serves of fruit and vegetables, lower intake of SSB, and regularly choosing wholegrain breads or cereals [16]. Reduction of fast food was targeted through the BCS but not accounted for in the HDHI subscore due to Covid-19 lockdowns/ ‘shelter in place’ mandates and the subsequent inability of participants to buy fast food during this time. The sum of the four items was taken to get a final HDHI subscore out of 16.

**Table 2:** Subscore of Healthy Diet Habits Index of behaviours targeted by behaviour change support

**Item**	**Target behaviour**	**Scoring**				
		0	1	2	3	4
**Fruit intake**	≥ 2 servings^c^ per day	None	0.5	1 per day	1.5	2 or more per day
**Vegetable intake**	≥ 5 servings^d^ per day	None	1	2 per day	3–4	5 or more per day
**Types of bread or cereals consumed**^**a**^	≥ 1 servings^e^ per day	Never choosing wholegrain options	-	Occasionally choosing wholegrain options (1–7 × per week)	-	Regularly choosing wholegrain options (> 7 per week)
**Sugar-sweetened beverages**^**b**^** intake**	≤ 1 250 ml glass per week	> 7 per week	5–6 per week	2–4 per week	1 per week or less	Never

^a^Wholegrain options in the Food Frequency Questionnaire include high fibre breakfast cereals (e.g. porridge, muesli, bran flakes, all bran), wholemeal or multigrain breads (including tortillas, pita, rolls, wraps), roti/chapatti, or brown rice and wholemeal pasta

^b^Sugar-sweetened beverage options in the Food Frequency Questionnaire include soft drinks, juices, cordials

^c^A serving of fruit is defined as 150 g

^d^A serving of vegetables is defined as 75 g

^e^A serving of wholegrains is equivalent to 40 g mixed grain bread

#### Barriers to healthy eating

To evaluate the effect of the BCS on participants’ self-perceived barriers to eating healthily, participants were required to fill in an online, 12-item questionnaire at baseline, week 5, and week 10. Each item was a succinct statement relating to a specific barrier and answered on a 4-point scale with the following response options: Strongly Disagree, Disagree, Agree, and Strongly Agree. The items for this questionnaire were developed based on influential barriers identified at step 2 of the Nine Principles framework, through relevant literature [25] and thematically analysed results from a target audience survey about barriers and enablers to healthy eating [20]. Together, this allowed identification of 12 key barriers that had a theoretical influence on attitude (e.g., apathy, knowledge, skills, time, cost, food environment), perceived behavioural control (e.g., low motivation and poor self-regulation), and subjective norms (e.g., social support) according to the Theory of Planned Behaviour [18]. Items were assessed for face validity and content validity by a second researcher on the team (RR) who has experience and expertise in BCTs and the dietary habits of young adults.

#### Adherence to dietary reporting

Participant entries in the Easy Diet Diary were assessed twice a week. Adherence to dietary reporting was defined as at least one full day of reporting (i.e., at least three main meals, or two meals and snacks) entered every 3–4 days. Participants received a score of one if they met the minimum requirements for reporting each week, or a score of zero if they failed to meet the requirements. The scores over the 10 weeks of the study were tallied to give a total score out of 10, which was then converted to a reporting score out of 100. This score was multiplied by their frequency of reporting (i.e., total meals reported divided by the maximum possible 210 meals reported over 10 weeks) to give a weighted reporting score.

#### Engagement with the facebook group

Active engagement is defined as interacting with Facebook, such as liking, commenting, or posting, while simply viewing posts is considered passive engagement [26]. Active engagement was retrospectively, objectively measured through manual count scores of likes and comments for each post per Facebook group. Passive engagement was measured through the percentage of participants in each group that reported had “seen” each post on Facebook. As each group contained less than 50 members, comprehensive Facebook analytics were not available.

#### Impact evaluation

At week 10 participants completed an online impact evaluation questionnaire that assessed their perception of the BCS. This consisted of six items on a 5-point Likert scale relating to components of the BCS such as the helpfulness of the Facebook group, text messages, and completing the intervention as a household pair. Each item had the following response options: Strongly Disagree, Disagree, Neutral, Agree, and Strongly Agree. Participants were asked two open-ended questions regarding what they liked and disliked about the nutrition support and why. Items were assessed for face and content validity by RR.

#### Statistical analysis

Analysis was performed on the data of the 78 participants who completed the study per protocol. An additional four participants were excluded from the analysis of barriers to healthy eating as they had missing data sets from at least one timepoint. One participant was missing a single response from a barrier to healthy eating item at baseline, so data was imputed using mean scores for that individual. Continuous data is reported as mean and standard deviation, and categorical data is presented as frequencies and percentages of votes for each item. Two-tailed paired t-tests were conducted to assess changes between baseline and week 10 for each component and the total score of the HDHI subscore for the total population. Linear mixed models were used to investigate if there was a difference in HDHI components for each intervention arm. For barriers to healthy eating questions, responses of ‘strongly disagree’ and ‘disagree’, or ‘strongly agree’ and ‘agree’, were aggregated for each item. Chi-square test for associations were then conducted for each item.

Logistic regression was used to assess differences in participant reporting scores according to the intervention group. The effect of possible combinations of covariates (demographic, health, and anthropometric characteristics gathered in the screening questionnaire) on reporting score was assessed using the corrected Akaike’s information criterion (AICc), with the model with the lowest AICc value chosen. The model was fitted as a mixed effects model, with intervention and the covariates chosen by the AICc as fixed effects (Covid-19 isolation, TFEQ) and couple (household pair) nested within cohort as random effects. Quantitative data was analysed using GraphPad Prism (GraphPad Software, Inc, version 9.4.1 (458)) and R 4.2.2. statistical software.

#### Thematic analysis

Open-ended responses from the impact evaluation were transcribed and entered into nVivo (release 1.5.2 (946)) for inductive qualitative analysis [27]. Steps involved initial familiarisation by reading the data, then systematically creating codes by identifying recurrent or meaningful features of the data. Codes were then compared and those that linked were clustered into themes with supporting data. All data scripts were read at least three times and moderated by a second researcher.

## Results

### Participant characteristics

A total of 78 participants completed the 10-week intervention (55% female, average age 25.8 ± 4.3 years, and well educated with 72% having achieved university-level education). Two participants withdrew from the study at week 5 of the intervention, with one individual of a pair experiencing gastrointestinal side effects. As the protocol requires participants to complete the study in a household unit, the other participant in this household was also discontinued.

### Change in dietary behaviours

There was a significant increase in the total HDHI subscore between baseline and week 10 (*p* < 0.05). Looking at the components of the score demonstrates this is driven by an increase in vegetable consumption (Table 3). Vegetable intake significantly increased by half a serve from baseline to the end of the 10-week dietary intervention (*p* < 0.001), shifting towards the government recommendations of 5 (female) – 6 (male) serves/day [16]. No difference in HDHI subscores between intervention groups was observed (see Additional file 2).

**Table 3:** Dietary behaviours from the Healthy Diet Habits Index subscore at baseline to week 10

**Dietary Behaviour**	**Baseline**	**Week 10**	***P* value**
Fruit (serves/day)^a^	1.6 ± 1.1	1.6 ± 1.0	0.950
Sugar-sweetened beverages (serves/day)^b^	0.6 ± 1.0	0.7 ± 1.2	0.441
Vegetables (serves/day)^c^	2.2 ± 1.1	2.8 ± 1.3	** < 0.001**
Wholegrains (serves/day)^d^	5.5 ± 4.6	5.9 ± 4.7	0.382
Total score out of 16	10.6 ± 2.6	11.2 ± 2.6	**0.011**

^a^A serving of fruit is defined as 150 g

^b^A serving of a sugar-sweetened beverage is 250 ml

^c^A serving of vegetables is defined as 75 g

^d^A serving of wholegrains is equivalent to 40 g mixed grain bread

Serving sizes based off the New Zealand Ministry of Health Eating and Activity Guidelines (2020)

### Barriers to healthy eating

After 10 weeks, the percentage of participants who disagreed with the statement “I don't have the knowledge required to eat a healthy diet” decreased from 16 to 5% (*p* = 0.025). There were no other significant changes in the perception of barriers. At baseline, the most prominent barriers to healthy eating included preferring the taste of unhealthy food (*n* = 95%), lack of self-control (*n* = 42%), and the high cost of eating healthily (*n* = 27%) (Table 4).

**Table 4:** Change in young adults perceived barriers to healthy eating from baseline to week 10

	**Baseline, n (%)**		**Week 5, n (%)**		**Week 10, n (%)**		***P* value**^**a**^
**Question**	**Disagree**	**Agree**	**Disagree**	**Agree**	**Disagree**	**Agree**	
I'm not interested in eating well	69 (93)	5 (7)	71 (96)	3 (4)	69 (93)	5 (7)	> 0.999
I don't have the knowledge required to eat a healthy diet	62 (84)	12 (16)	69 (93)	5 (7)	70 (95)	4 (5)	**0.025**
The people I live with do not support me when I try to eat well	66 (89)	8 (11)	68 (92)	6 (8)	67 (91)	7 (9)	0.779
Eating well is not important to me	70 (95)	4 (5)	67 (91)	7 (9)	68 (92)	6 (8)	0.536
Lack of self-control prevents me from eating a healthy diet	43 (58)	31 (42)	46 (62)	28 (38)	44 (59)	30 (41)	0.867
I have no motivation to eat well	69 (93)	5 (7)	70 (95)	4 (5)	72 (97)	2 (3)	0.256
I prefer the taste of unhealthy food	4 (5)	70 (95)	0 (0)	74 (100)	3 (4)	71 (96)	0.638
My skills in preparing/cooking healthy food prevent me from eating well	66 (89)	8 (11)	66 (89)	8 (11)	69 (93)	5 (7)	0.399
There are no healthy options around for me to choose from	68 (92)	6 (8)	71 (96)	3 (4)	72 (97)	2 (3)	0.130
I don't have the facilities required to cook and eat a healthy diet	73 (99)	1 (1)	72 (97)	2 (3)	72 (97)	2 (3)	NA^b^
It costs too much money to eat a healthy diet	54 (73)	20 (27)	50 (68)	24 (32)	52 (70)	22 (30)	0.719
I don’t have enough time to eat well	57 (77)	17 (23)	55 (74)	19 (26)	51 (69)	23 (31)	0.264

^a^Statistical significance (*P* < 0.05) from baseline to week 10. Significance tested by chi-square test for trend

^b^Inappropriate to perform chi-square test for trend as less than 20% of the expected values are greater than 5

### Adherence to dietary reporting

**Fig. 1:**
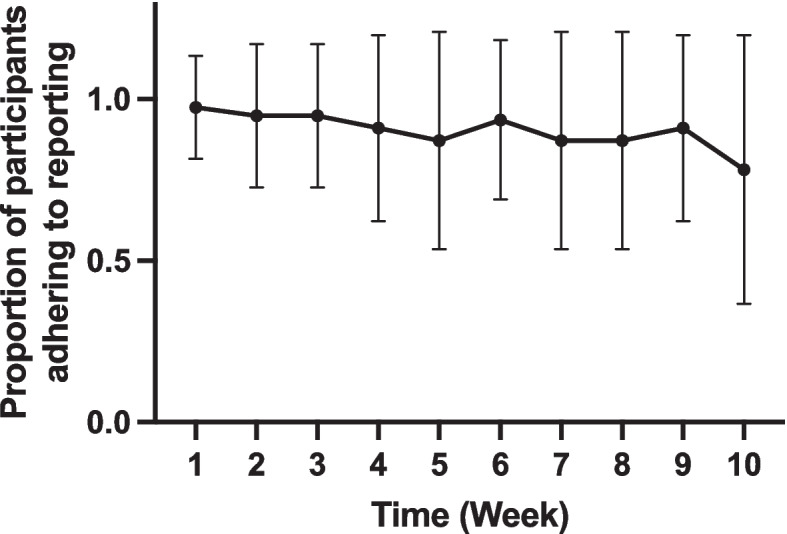
Proportion of total participants adhering to reporting requirements each week. Error bars represent standard deviation

Overall adherence to reporting was high, with adherence scores that could range from 0 – 100, mean reporting score of the total population was 90.4 ± 14.6, while the weighted reporting score was 86.0 ± 16.8. This remained relatively stable across the 10 weeks, dropping off slightly in week 10 with greater variability in scores (Fig. 1).

Participants who had to isolate during the intervention due to being in a household that contracted Covid-19 were 74% less likely to meet reporting requirements (Table 5), although this effect of isolation was no longer significant when adherence scores were weighted on the maximum number of reporting opportunities. The TFEQ looks at eating restraint, disinhibition, and emotional eating, and gave an indication of baseline eating behaviours [22]; this eating behaviour score of the participant did not influence dietary reporting, nor did intervention group.

**Table 5:** Outcome measures derived from logistic mixed effect models comparing differences in reporting scores between intervention arms

	**Estimate (SE)**	***t***	***df***	***P* value**
**Adherence – Reporting**				
Vegetarian group	-0.57	-0.87	7.23	0.4133
Covid-19 isolation	-1.34	-2.81	41.62	**0.0076**
TFEQ score^a^	-1.98	-1.11	67.26	0.2701
**Adherence – Reporting Weighted**				
Vegetarian group	-0.15	-0.23	6.94	0.8264
Covid-19 isolation	-0.73	-1.44	41.75	0.1571
TFEQ score^a^	-2.56	-1.39	65.23	0.1697

^a^*TFEQ* Three-factor eating questionnaire at baseline

### Engagement with the facebook group

There were 29–31 Facebook posts from researchers for the participants in Groups 1 and 2. Some post content was transitioned to deliver via Facebook Messenger for Group 3–8, so these latter groups received 19–21 posts. Groups 4, 5, and 8 received two more posts than Groups 3, 6, 7a, and 7b due to diet allocation. Active and passive engagement was relatively low over the 10 weeks (Table 6).

**Table 6:** Engagement data for Facebook groups within the PREDITION trial

**Group**	**Intervention**	**Number in group**	**Reacts**^**a**^** (n)**	**Comments (n)**	**Participant posts (n)**	**Seen**^**b**^** (%)**
**1**	Flexitarian	9	24	12	4	86
**2**	Vegetarian	9	4	4	1	69
**3**	Vegetarian	8	4	0	0	68
**4**	Flexitarian	10	16	0	0	50
**5**	Flexitarian	10	19	5	0	72
**6**	Vegetarian	10	12	2	1	75
**7a**	Vegetarian	4	2	1	0	33
**7b**	Vegetarian	5	7	0	0	84
**8**	Flexitarian	10	3	0	0	47

^a^Reacts include a count score with a count given to any participant ‘reactions’ to a post e.g. selecting ‘likes’, ‘loves’, ‘laughs’ reacts

^b^Seen refers to the average percentage of participants in each group who viewed each post

### Impact evaluation

Most participants agreed or strongly agreed that receiving text messages (*n* = 81%) and being accountable to a dining partner (i.e., their household pair; *n* = 92%) helped them to adhere to dietary reporting requirements and that the Facebook group helped them adhere to eating a healthy diet (*n* = 77%; Table 7). Overall, most participants agreed or strongly agreed that social media support helped alleviate barriers to participating in this trial (*n* = 68%).

**Table 7:** Quantitative responses to impact evaluation of behaviour change support components

**Question**	**Strongly disagree, n (%)**	**Disagree, n (%)**	**Neutral, n (%)**	**Agree, n (%)**	**Strongly Agree, n (%)**
I was satisfied with the Woop meals provided to me during the study	0 (0)	2 (3)	4 (5)	24 (31)	48 (62)
I was satisfied with the plant-based meat alternatives/red meat provided to me during the study	4 (5)	7 (9)	8 (10)	20 (26)	39 (50)
I enjoyed eating the plant-based meat alternatives/red meat provided during this study	7 (9)	8 (10)	8 (10)	23 (29)	32 (41)
It was easy for me to adhere to a predominantly vegetarian diet with some plant-based meat alternatives/red meat	0 (0)	2 (3)	6 (8)	34 (44)	36 (46)
I am very likely to continue to eat a predominantly vegetarian diet in the future	7 (9)	12 (15)	15 (19)	16 (21)	28 (36)
I am very likely to continue to eat a moderate amount of red meat/plant-based meat alternatives in the future	7 (9)	9 (12)	10 (13)	19 (24)	33 (42)
It was easy for me to record all my food intake using the Easy Diet Diary app	2 (3)	7 (9)	11 (14)	23 (29)	35 (45)
The text messages helped me to record my food intake on the Easy Diet Diary app	1 (1)	3 (4)	11 (14)	22 (28)	41 (53)
Being accountable to my dining partner helped me stick to my diet and record my food intake	0 (0)	1 (1)	5 (6)	35 (45)	37 (47)
The Facebook group helped me to stick to eating a healthy diet	5 (6)	5 (6)	8 (10)	19 (24)	41 (53)
The structure and format of the social media nutrition support was excellent	2 (3)	6 (8)	7 (9)	27 (35)	36 (46)
The social media nutrition support helped alleviate any barriers to participating in this dietary intervention trial	3 (4)	7 (9)	15 (19)	20 (26)	33 (42)

Aspects of the social media support that participants found helpful included food ideas, healthy eating education, consistent support and connection, and reminders helpful (Table 8). When asked what was unhelpful a common theme was that many participants did not engage with it, with reasons being due to not using social media in general or not having met other group members (Table 9). The lack of specific, tailored advice was also another unhelpful aspect.

**Table 8:** Components of the social media nutrition support participants found helpful

**Theme**	**Description**	**Example Participant Quotes**
**Food ideas**	Participants found the recipes, cooking videos and snack ideas helpful for cooking meals that aligned with the intervention	*It was nice to see posts with inspiration for meals that were healthy and vegetarian* *The recipe ideas were particularly helpful*
**Healthy eating education**	Participants found the educational tips and videos on healthy eating useful and motivating	*The presentation videos at the start of each block. I appreciated the broader coverage of topics which were based on up-to-date evidence* *It was useful to get some trustworthy information about diet, as there's a lot of contradicting and sometimes unsafe tips online*
**Consistent support and connection**	Participants found it helpful having a point of connection and group where they could ask questions	*Knowing i could ask questions if i needed to* *It was good to see that there were other people who were also taking part in the study, and hearing their thoughts on it* *It is good to have support and group*
**Reminders**	Participants found it useful as a reminder/nudge to eat healthily	*Good reminders of little steps to improve healthy eating* *Helpful updates and tips to keep us on track*

**Table 9:** Components of the social media nutrition support that participants did not find helpful

**Theme**	**Description**	**Example Participant Quotes**
**Lack of engagement**	Many participants commented that they did not engage with it, either because they didn’t use social media in general or did not feel connected to other participants	*I didn't engage very much with the social media nutrition support* *Don't use social media* *engagement was limited with the participants; possibly due to not knowing the others personally*
**Not tailored enough**	Some participants found the content was not tailored enough for their needs and level of nutrition knowledge/ skills	*Some of the information was very basic, to me, and not helpful. But for others I could see it being helpful* *It was not specific enough, it was aimed primarily at people who are not as informed with healthy diets*
**Nothing was unhelpful**	Many participants reported nothing was unhelpful about the nutrition support, but it did not add value to them	*Wasn’t unhelpful, just didn’t add a lot of value* *Nothing—just not really my way of learning about things*

## Discussion

This article evaluates how effective the BCS was at enhancing participant adherence to the study-related behaviours of (i) eating a healthy basal diet and (ii) dietary reporting using a smartphone app within the PREDITION trial. Adherence to a healthy diet was important for elucidating the effects of moderate lean red meat consumption in the context of a balanced diet, the PREDITION trial’s primary aim. The relatively high baseline HDHI subscore demonstrated a small but significant increase at week 10, indicating a healthy basal diet was achieved and maintained. High adherence to reporting scores were also observed throughout the study. This strengthened study validity by allowing us to identify if participants complied with study requirements of consuming the intervention protein within a vegetarian diet.

To date, behaviour change frameworks have predominantly been used to design interventions where the primary aim is behaviour change itself [28, 29], as opposed to supporting study-related behaviours, as done here. Recently, there has been movement in this field towards using behaviour change frameworks for this latter purpose, with behaviour change theory being used to enhance adherence to online dietary interventions [30]. Similarly, there are an emerging number of studies proposing how behaviour change theory can be used to enhance trial-related behaviours, such as recruiting participants [31] and returning questionnaires [32]; the efficacy of these interventions is yet to be tested [12]. To the best of our knowledge, this is the first study to provide an example of how BCS can be systematically implemented and assessed for improving adherence to behaviours required within a nutrition trial using the Nine Principles framework.

Using behaviour change frameworks to design interventions frequently results in utilising a combination of BCTs and modes of delivery [33], as seen in our BCS (Table 1). Hence, considering the efficacy of different BCS components is key for aiding researchers in selecting effective BCTs and modes of delivery for future research. For instance, the significant increase in HDHI subscore between baseline and week 10 was mostly driven by an increase in vegetable consumption. Increasing vegetable intake was targeted through multiple BCTs within the BCS, including ‘Adding objects to the environment’, ‘Information about health consequences’, ‘Instruction on how to perform a behaviour’, and ‘Credible source’. Although the impact of these BCTs could not be assessed independently, increasing fruit intake was also targeted by the same BCTs except for ‘Adding objects to the environment’. However, participants did not increase their fruit intake. In agreement with results of a previous systematic review [34], this suggests that the BCT ‘Adding objects to the environment’ is likely to be an effective BCT for improving vegetable intake of young adults.

A published review on the efficacy of behavioural interventions has reported modest improvements on healthy eating indices, although what is considered a clinically significant improvement is unclear [35]. Hence, while we report a statistically significant change in healthy eating before and after, the clinical relevance of the change may be small. Low participant engagement with the Facebook groups, as indicated by social media metrics and impact evaluation, is likely to play a role in the observed change. For BCTs to be effective, participants must have both exposure to, and engagement with them [36, 37]. Engagement is complex and has been conceptualised as the extent of usage as well as the subjective experience of the user, including attention, interest, and affect [38]. Other social media-based interventions have commonly employed similar BCTs to those delivered by Facebook here [39]; however, as effectiveness of the selected BCTs is limited by the extent of engagement [38], it is difficult to draw a conclusion on their effectiveness. This highlights the importance of identifying and using acceptable modes of delivery during BCS development.

Lack of motivation may also explain the low engagement and small change in HDHI subscore [40]. Possible reasons for this include participants’ belief that their diet was healthy enough at baseline [41] or due to lack of tailored support, which has been shown to maximise user engagement [36] and improve effectiveness of computer-tailored nutrition interventions [42]. Sustaining engagement with eHealth interventions long enough to establish behaviour change is a commonly acknowledged challenge [43]. Greater reporting on engagement with behaviour change components of trials, including quantitative and qualitative measures, would improve our understanding of how engagement can be enhanced [36, 44], especially as reporting on user metrics alone may not accurately reflect engagement with the behaviour change process itself [45].

Alongside changes in dietary intake towards a healthy eating profile, the reporting of dietary intake was good throughout the trial, a behaviour that is consistently trying to be achieved within nutrition trials [46]; albeit there were lower levels of reporting and more variability as the trial progressed, as seen in previous research [47]. As with healthy eating, a combination of BCTs resulted in high adherence to reporting requirements over the 10 weeks, a behaviour required for participant retention within the PREDITION trial. A Cochrane review of strategies to increase retention in randomised trials highlighted how monetary incentives improved questionnaire response rates [48]. Indeed, rewards, along with electronic prompts are often implicitly incorporated into trials and have further evidence of enhancing participant retention [10]. The meal kits participants were provided had a recognisable monetary value, and the threat of removing this reward may have sufficiently motivated participants to adhere to reporting requirements. Rewards can be effective at initiating behaviour change but may not sustain behaviour after removal of the reward [49]. The use of prompts/cues and feedback delivered via text message was also likely to play a key role in supporting this behaviour [50], as well as consistent contact between researchers and participants [51]. Identifying BCTs that supplement the more resource-intensive BCTs like rewards is important to ensure feasibility of the BCS.

Strengths of this evaluation include the use of a specified taxonomy of BCTs which enabled clear identification of the active components within the BCS [11]. We considered engagement with the modes of delivery through quantitative metrics, a previously overlooked aspect of behavioural interventions [44]. Additionally, we directly assessed adherence to the target behaviours themselves, as opposed to proxy measures of the behaviour. For example, it is common for weight management interventions to assess adherence through attrition or attendance to intervention sessions which does not sufficiently capture dietary behaviour change [52]. Transparent and detailed documentation of the development and fidelity of support within a trial is essential for furthering the field of behaviour change science [51, 53]. The wider implications of this include enabling optimisation of large-scale public health campaigns.

A key limitation of this evaluation is that there was no control group, with all participants receiving BCS. This means we cannot be certain that improvements in the target behaviours were due to the BCS. For instance, the small change in the relatively healthy baseline dietary behaviours could be due to outside factors, such as a change in the price of vegetables. Additionally, adherence to eating behaviours was measured through a self-reported FFQ which is subject to self-reporting biases [4]. Future research would benefit from conducting a study within a trial investigating adherence to study requirements using systematically designed BCS compared to no BCS, or usual practice. However, this would be challenging given that BCTs are frequently implicitly used within trials to help both intervention and control groups adhere to requirements, and what constitutes ‘usual practice’ is not yet documented and likely to vary between researchers [10, 39]. Furthermore, the feasibility of using the Nine Principles framework in this capacity was not assessed. As researchers frequently have limited time and resources, future research should investigate the feasibility of different behaviour change frameworks, such as the Behaviour Change Wheel [33], or simply components of frameworks, for the purpose of enhancing adherence to trial-related behaviours.

## Conclusion

The evaluation of this BCS as a whole program provides promising results that using a behaviour change framework to systematically design support can promote adherence to study requirements within a nutrition trial. Using behaviour change science in this way to enhance the validity of research is an innovative and emerging idea and further research into the feasibility and effectiveness of using of different behaviour change frameworks for this purpose is warranted.

### Supplementary Information

**Additional file 1. **Completed Template for Intervention Description and Replication framework.

**Additional file 2: Table A.** Healthy Diet Habits Index subscore changes from baseline to week 10 according to dietary intervention.

**Additional file 3. **Reporting checklist for randomised trial.

## Data Availability

The datasets used and/or analysed during the current study are available from the corresponding author on reasonable request.

## Abbreviations

AICc: Akaike’s Information Criterion
BCS: Behaviour Change Support
BCT: Behaviour Change Technique
eHealth: Electronic Health
HDHI: Healthy Diet Habits Index
PREDITION: Protein Diet Satisfaction
SSB: Sugar Sweetened Beverage
TFEQ: Three Factor Eating Questionnaire
