# Supplementary material for: Implementing multicomponent, eHealth-based behaviour change support within a dietary intervention trial improves adherence to study-related behaviours in healthy young adults
Source: BMC Nutr. 2023 Nov 21;9:134. doi: 10.1186/s40795-023-00798-7 (PMC10664496; doi:10.1186/s40795-023-00798-7)
Supplement: Supplementary file 2 — Additional file 2: Table A. Healthy Diet Habits Index subscore changes from baseline to week 10 according to dietary intervention. [file 40795_2023_798_MOESM2_ESM.docx]

**Table A.** Healthy Diet Habits Index subscore changes from baseline to week 10 according to dietary intervention.

|  | Total population | |  | Flexitarian (n=40) | |  | Vegetarian (n=38) | |  | P values | | |
| --- | --- | --- | --- | --- | --- | --- | --- | --- | --- | --- | --- | --- |
|  | Baseline | Week 10 |  | Baseline | Week 10 |  | Baseline | Week 10 |  | Time | Diet | Time x Diet |
| Fruit (serves/day) | 1.6 ± 1.1 | 1.6 ± 1.0 |  | 1.6 ± 1.1 | 1.5 ± 0.9 |  | 1.7 ± 1.1 | 1.7 ± 1.1 |  | 0.937 | 0.474 | 0.640 |
| Sugar-sweetened beverages (serves/day) | 0.6 ± 1.0 | 0.7 ±1.2 |  | 0.6 ±1.1 | 0.8 ±1.4 |  | 0.6 ±0.9 | 0.5 ±0.9 |  | 0.795 | 0.470 | 0.357 |
| Vegetables (serves/day) | 2.2 ± 1.1 | 2.8 ± 1.3 |  | 2.2 ± 1.4 | 2.7 ± 1.5 |  | 2.1 ± 0.9 | 2.9 ± 1.1 |  | **<0.001** | 0.756 | 0.305 |
| Wholegrains (serves/day) | 5.5 ± 4.6 | 5.9 ± 4.7 |  | 5.9 ±4.1 | 6.2 ± 4.3 |  | 5.1 ± 5.1 | 5.6 ± 5.0 |  | 0.357 | 0.465 | 0.774 |
| Total score | 10.6 ± 2.6 | 11.2 ±2.6 |  | 10.6 ±2.6 | 10.9 ± 2.6 |  | 10.5 ± 2.6 | 11.4 ± 2.6 |  | **0.026** | 0.684 | 0.335=6 |
