# Supplementary material for: Implementing multicomponent, eHealth-based behaviour change support within a dietary intervention trial improves adherence to study-related behaviours in healthy young adults
Source: BMC Nutr. 2023 Nov 21;9:134. doi: 10.1186/s40795-023-00798-7 (PMC10664496; doi:10.1186/s40795-023-00798-7)
Supplement: Supplementary file 3 — Additional file 3. Reporting checklist for randomised trial. [file 40795_2023_798_MOESM3_ESM.docx]

# Reporting checklist for randomised trial.

Based on the CONSORT guidelines.

## Instructions to authors

Complete this checklist by entering the page numbers from your manuscript where readers will find each of the items listed below.

Your article may not currently address all the items on the checklist. Please modify your text to include the missing information. If you are certain that an item does not apply, please write "n/a" and provide a short explanation.

Upload your completed checklist as an extra file when you submit to a journal.

In your methods section, say that you used the CONSORTreporting guidelines, and cite them as:

Schulz KF, Altman DG, Moher D, for the CONSORT Group. CONSORT 2010 Statement: updated guidelines for reporting parallel group randomised trials

|  |  | Reporting Item | Page Number |
| --- | --- | --- | --- |
| **Title and Abstract** |  |  |  |
| Title | [#1a](https://www.goodreports.org/reporting-checklists/consort/info/#1a) | Identification as a randomized trial in the title. | n/a the behaviour change support was implemented WITHIN a randomised trial but not a randomised trial itself (i.e. it was applied across both arms) |
| Abstract | [#1b](https://www.goodreports.org/reporting-checklists/consort/info/#1b) | Structured summary of trial design, methods, results, and conclusions | 1-2 |
| **Introduction** |  |  |  |
| Background and objectives | [#2a](https://www.goodreports.org/reporting-checklists/consort/info/#2a) | Scientific background and explanation of rationale | 3-5 |
| Background and objectives | [#2b](https://www.goodreports.org/reporting-checklists/consort/info/#2b) | Specific objectives or hypothesis | 6 |
| **Methods** |  |  |  |
| Trial design | [#3a](https://www.goodreports.org/reporting-checklists/consort/info/#3a) | Description of trial design (such as parallel, factorial) including allocation ratio. | 5 |
| Trial design | [#3b](https://www.goodreports.org/reporting-checklists/consort/info/#3b) | Important changes to methods after trial commencement (such as eligibility criteria), with reasons | 6 |
| Participants | [#4a](https://www.goodreports.org/reporting-checklists/consort/info/#4a) | Eligibility criteria for participants | 6-7 |
| Participants | [#4b](https://www.goodreports.org/reporting-checklists/consort/info/#4b) | Settings and locations where the data were collected | 6 |
| Interventions | [#5](https://www.goodreports.org/reporting-checklists/consort/info/#5) | The experimental and control interventions for each group with sufficient details to allow replication, including how and when they were actually administered | 5 (and Table 1) |
| Outcomes | [#6a](https://www.goodreports.org/reporting-checklists/consort/info/#6a) | Completely defined prespecified primary and secondary outcome measures, including how and when they were assessed | 7-11 |
| Sample size | [#7a](https://www.goodreports.org/reporting-checklists/consort/info/#7a) | How sample size was determined. | n/a Sample size calculation is conducted and reported in the protocol for the randomised trial the behaviour change support was implemented in (Braakhuis A, Gillies N, Worthington A, Knowles S, Conner T, Roy R, et al. A Modern Flexitarian Dietary Intervention Incorporating Web-Based Nutrition Education in Healthy Young Adults: Protocol for a Randomized Controlled Trial. JMIR Res Protoc [Internet]. 2021 Dec 21;10(12):e30909. Available from: https://www.researchprotocols.org/2021/12/e30909) |
| Sample size | [#7b](https://www.goodreports.org/reporting-checklists/consort/info/#7b) | When applicable, explanation of any interim analyses and stopping guidelines | n/a no interim analyses or stopping guidelines |
| Randomization - Sequence generation | [#8a](https://www.goodreports.org/reporting-checklists/consort/info/#8a) | Method used to generate the random allocation sequence. |  |
| n/a no randomisation was completed for the behaviour change support which was the focus of this article. |  |  |  |
| Randomization - Sequence generation | [#8b](https://www.goodreports.org/reporting-checklists/consort/info/#8b) | Type of randomization; details of any restriction (such as blocking and block size) |  |
| n/a no randomisation was completed for the behaviour change support which was the focus of this article. |  |  |  |
| Randomization - Allocation concealment mechanism | [#9](https://www.goodreports.org/reporting-checklists/consort/info/#9) | Mechanism used to implement the random allocation sequence (such as sequentially numbered containers), describing any steps taken to conceal the sequence until interventions were assigned | n/a no randomisation was completed for the behaviour change support which was the focus of this article. |
| Randomization - Implementation | [#10](https://www.goodreports.org/reporting-checklists/consort/info/#10) | Who generated the allocation sequence, who enrolled participants, and who assigned participants to interventions | n/a no randomisation was completed for the behaviour change support which was the focus of this article. |
| Blinding | [#11a](https://www.goodreports.org/reporting-checklists/consort/info/#11a) | If done, who was blinded after assignment to interventions (for example, participants, care providers, those assessing outcomes) and how. | n/a no blinding was involved as all participants received behaviour change support. |
| Blinding | [#11b](https://www.goodreports.org/reporting-checklists/consort/info/#11b) | If relevant, description of the similarity of interventions | n/a no blinding was involved as all participants received behaviour change support. |
| Statistical methods | [#12a](https://www.goodreports.org/reporting-checklists/consort/info/#12a) | Statistical methods used to compare groups for primary and secondary outcomes | 10-11 |
| Statistical methods | [#12b](https://www.goodreports.org/reporting-checklists/consort/info/#12b) | Methods for additional analyses, such as subgroup analyses and adjusted analyses | 10-11 |
| Outcomes | [#6b](https://www.goodreports.org/reporting-checklists/consort/info/#6b) | Any changes to trial outcomes after the trial commenced, with reasons | n/a as there were no changes to trial outcomes |
| **Results** |  |  |  |
| Participant flow diagram (strongly recommended) | [#13a](https://www.goodreports.org/reporting-checklists/consort/info/#13a) | For each group, the numbers of participants who were randomly assigned, received intended treatment, and were analysed for the primary outcome | n/a this is not relevant for evaluation of the behaviour change support as all participant received it. |
| Participant flow | [#13b](https://www.goodreports.org/reporting-checklists/consort/info/#13b) | For each group, losses and exclusions after randomization, together with reason | 11 |
| Recruitment | [#14a](https://www.goodreports.org/reporting-checklists/consort/info/#14a) | Dates defining the periods of recruitment and follow-up | n/a full recruitment details will be published with the primary outcomes of the randomised trial that the behaviour change support was implemented in (in publication elsewhere) |
| Recruitment | [#14b](https://www.goodreports.org/reporting-checklists/consort/info/#14b) | Why the trial ended or was stopped | n/a full recruitment details will be published with the primary outcomes of the randomised trial that the behaviour change support was implemented in (in publication elsewhere) |
| Baseline data | [#15](https://www.goodreports.org/reporting-checklists/consort/info/#15) | A table showing baseline demographic and clinical characteristics for each group | 11 (relevant demographics presented in text not table) |
| Numbers analysed | [#16](https://www.goodreports.org/reporting-checklists/consort/info/#16) | For each group, number of participants (denominator) included in each analysis and whether the analysis was by original assigned groups | 10 |
| Outcomes and estimation | [#17a](https://www.goodreports.org/reporting-checklists/consort/info/#17a) | For each primary and secondary outcome, results for each group, and the estimated effect size and its precision (such as 95% confidence interval) | 12-14 |
| Outcomes and estimation | [#17b](https://www.goodreports.org/reporting-checklists/consort/info/#17b) | For binary outcomes, presentation of both absolute and relative effect sizes is recommended | n/a no binary outcomes reported |
| Ancillary analyses | [#18](https://www.goodreports.org/reporting-checklists/consort/info/#18) | Results of any other analyses performed, including subgroup analyses and adjusted analyses, distinguishing pre-specified from exploratory | Additional file 2 |
| Harms | [#19](https://www.goodreports.org/reporting-checklists/consort/info/#19) | All important harms or unintended effects in each group (For specific guidance see CONSORT for harms) | 11 |
| **Discussion** |  |  |  |
| Limitations | [#20](https://www.goodreports.org/reporting-checklists/consort/info/#20) | Trial limitations, addressing sources of potential bias, imprecision, and, if relevant, multiplicity of analyses | 21-22 |
| Interpretation | [#22](https://www.goodreports.org/reporting-checklists/consort/info/#22) | Interpretation consistent with results, balancing benefits and harms, and considering other relevant evidence | 19-21 |
| Registration | [#23](https://www.goodreports.org/reporting-checklists/consort/info/#23) | Registration number and name of trial registry | 3 |
| Generalisability | [#21](https://www.goodreports.org/reporting-checklists/consort/info/#21) | Generalisability (external validity, applicability) of the trial findings | 22 |
| **Other information** |  |  |  |
| Interpretation | [#22](https://www.goodreports.org/reporting-checklists/consort/info/#22) | Interpretation consistent with results, balancing benefits and harms, and considering other relevant evidence | 19-21 |
| Registration | [#23](https://www.goodreports.org/reporting-checklists/consort/info/#23) | Registration number and name of trial registry | 3 |
| Protocol | [#24](https://www.goodreports.org/reporting-checklists/consort/info/#24) | Where the full trial protocol can be accessed, if available | 5 |
| Funding | [#25](https://www.goodreports.org/reporting-checklists/consort/info/#25) | Sources of funding and other support (such as supply of drugs), role of funders | 23 |

Notes:

- 1a: n/a the behaviour change support was implemented WITHIN a randomised trial but not a randomised trial itself (i.e. it was applied across both arms)
- 5: 5 (and Table 1)
- 7a: n/a Sample size calculation is conducted and reported in the protocol for the randomised trial the behaviour change support was implemented in (Braakhuis A, Gillies N, Worthington A, Knowles S, Conner T, Roy R, et al. A Modern Flexitarian Dietary Intervention Incorporating Web-Based Nutrition Education in Healthy Young Adults: Protocol for a Randomized Controlled Trial. JMIR Res Protoc [Internet]. 2021 Dec 21;10(12):e30909. Available from: https://www.researchprotocols.org/2021/12/e30909)
- 7b: n/a no interim analyses or stopping guidelines
- 8a: n/a no randomisation was completed for the behaviour change support which was the focus of this article.
- 8b: n/a no randomisation was completed for the behaviour change support which was the focus of this article.
- 9: n/a no randomisation was completed for the behaviour change support which was the focus of this article.
- 10: n/a no randomisation was completed for the behaviour change support which was the focus of this article.
- 11a: n/a no blinding was involved as all participants received behaviour change support.
- 11b: n/a no blinding was involved as all participants received behaviour change support.
- 13a: n/a this is not relevant for evaluation of the behaviour change support as all participant received it.
- 14a: n/a full recruitment details will be published with the primary outcomes of the randomised trial that the behaviour change support was implemented in (in publication elsewhere)
- 14b: n/a full recruitment details will be published with the primary outcomes of the randomised trial that the behaviour change support was implemented in (in publication elsewhere)
- 15: 11 (relevant demographics presented in text not table)
- 17b: n/a no binary outcomes reported
- 18: Additional file 2
- 6b: n/a as there were no changes to trial outcomes The CONSORT checklist is distributed under the terms of the Creative Commons Attribution License CC-BY. This checklist was completed on 09. March 2023 using <https://www.goodreports.org/>, a tool made by the [EQUATOR Network](https://www.equator-network.org) in collaboration with [Penelope.ai](https://www.penelope.ai)
